# Deciphering O‑GlcNAc-Dependent Signaling Via Integrated Proteomics and Phosphoproteomics

**DOI:** 10.1021/acsomega.6c02310

**Published:** 2026-06-04

**Authors:** Ci Wu, Chunyan Hou, Xinyue Wang, Yihan Peng, Yao Lin, Stephen W. Byers, Huadong Pei, Junfeng Ma

**Affiliations:** † Department of Oncology, Lombardi Comprehensive Cancer Center, 12231 Georgetown University Medical Center, Washington, District of Columbia 20007, United States; ‡ School of Chemistry and Chemical Engineering, 66523 Liaoning Normal University, Dalian 116029, China

## Abstract

Post-translational modifications (PTMs) on proteins play crucial roles in various biological processes. Two highly dynamic modifications, phosphorylation and O-linked N-acetylglucosamine modification (O-GlcNAcylation), are essential for cellular physiology and pathology. Emerging evidence suggests intimate crosstalk between phosphorylation and O-GlcNAcylation on multiple proteins. However, the precise nature of their crosstalk remains largely unknown. In this study, we explored the crosstalk between phosphorylation and O-GlcNAcylation using the pancreatic ductal cell line PANC-1 as a model. Proteome and phosphoproteome changes were measured for cells treated with OSMI-1, a specific inhibitor of O-GlcNAc transferase, and Thiamet G, a specific inhibitor of O-GlcNAcase. Among the 8938 phosphorylation sites quantified, 2289 phosphosites on 1225 proteins and 2201 phosphosites on 1199 proteins were significantly altered by OSMI-1 and TMG treatment, respectively, demonstrating extensive crosstalk between O-GlcNAcylation and phosphorylation. Further analysis revealed widespread phosphorylation changes of the kinome and phosphatome, even after a short-term perturbation with inhibitors to O-GlcNAc cycling enzymes. Moreover, phosphoproteomic profiling, kinase inhibition experiments, and in vitro kinase assays identified that phosphorylation of OGA itself at S364 is specifically mediated by casein kinase 2 α (CK2α). These results uncover glycosylation-dependent cellular signaling through the potentially multilayer crosstalk between phosphorylation and O-GlcNAcylation.

## Graphical abstract

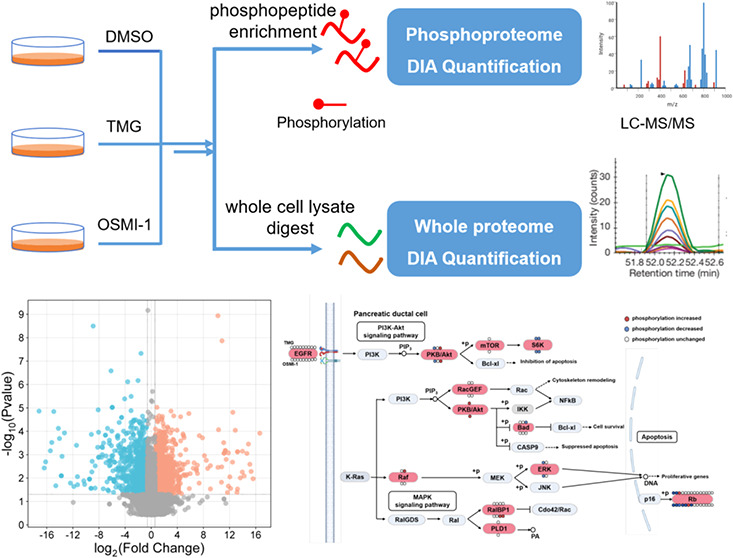

## Introduction

Post-translational modifications (PTMs) decorate cellular proteins, regulating their structure, interactions, and ultimately their function. Among these, phosphorylation and O-linked β-N-acetylglucosamine modification (O-GlcNAcylation) are two highly dynamic PTMs that play key roles in cellular physiology and pathology.^1–3^ O-GlcNAcylation is a reversible modification in which a single β-N-acetylglucosamine (GlcNAc) molecule is added to the hydroxyl group of serine, threonine, and tyrosine residues on target proteins.^3,4^ Like phosphorylation, O-GlcNAcylation is a rapidly cycling process, with O-GlcNAc transferase (OGT) regulating its addition and O-GlcNAcase (OGA) controlling its removal. In contrast, phosphorylation is catalyzed by a plethora of kinases and phosphatases.^5,6^

Given that O-GlcNAcylation targets the same amino acids as phosphorylation, the crosstalk between these two PTMs has become an important field.^7–10^ Phosphorylation and O-GlcNAcylation crosstalk can manifest in multiple ways, including direct competition for the same amino acid residue (reciprocal crosstalk) as well as through modifications that influence each other at adjacent or even distant sites on the protein sequence.^9–12^ Isotopic labeling-based high-throughput proteomics showed that acutely increasing O-GlcNAcylation by treatment with PUGNAc and NAG-thiazoline (two inhibitors of poor selectivity toward OGA) decreased phosphorylation at 280 sites and increased phosphorylation at 148 sites of proteins in NIH-3T3 cells.^13^ Further low-throughput studies show that this interplay occurs not only on many protein substrates but also on enzymes responsible for adding these modifications (i.e., kinases and OGT)^14–19^ and those that remove them (i.e., phosphatases and OGA),^20–22^ regulating protein activity, localization, and stability. Mass spectrometry-based approaches coupled with *in vitro* assays were also used to explore potential crosstalk mechanisms, uncovering a few motifs that may govern the reciprocal regulation (e.g., obstructed O-GlcNAcylation at sites phosphorylated by proline-directed kinases).^23^ Moreover, overexpression of OGT or deletion of OGT also induced substantial phosphorylation changes in different types of cells.^12,24^ Despite growing interest in the crosstalk between these two modifications, major knowledge gaps remain, particularly regarding the global effects of modulating O-GlcNAcylation on proteomes and phosphoproteomes. More importantly, pharmacological inhibition of OGT/OGA has been recently proposed as a promising therapeutic strategy in diseases such as cancer and neurodegeneration.^11,25–28^ However, how OGT/OGA inhibition would rewire signaling networks at the (phospho)­proteome level remains poorly understood. The lack of quantitative and systems-level studies limits our ability to predict downstream consequences of OGT/OGA-targeted therapies. Therefore, comprehensive profiling of (phospho)­proteome changes under OGT/OGA inhibition holds the potential to facilitate a better understanding of the mechanistic outcomes and potential effects of such interventions.

Here, we explored the crosstalk between phosphorylation and O-GlcNAcylation by systematic analysis of (phospho)­proteomics of PANC-1 cells treated with OSMI-1 (a potent and specific inhibitor of OGT) and thiamet G (TMG, a potent and specific inhibitor of OGA). Besides phosphorylation changes on many substrate proteins, we investigated the effects of OGA and OGT inhibition on the status of enzymes directly involved in protein phosphorylation and O-GlcNAcylation cycling (i.e., protein kinases, phosphatases, OGT, and OGA). This study not only provides comprehensive and quantitative data sets of phosphorylation regulated by disrupted O-GlcNAcylation but also provides deep insights into our understanding of the crosstalk between protein phosphorylation and O-GlcNAcylation.

## Experimental Section

### Phosphopeptide Enrichment by TiO_2_ Beads

Three hundred micrograms of the PANC-1 protein digests were used for phosphopeptide enrichment by using the TiO_2_ spin tip (GL Sciences) according to the manufacturer’s instructions. TiO_2_ spin tip (200 μL) was first conditioned by adding 200 μL of buffer A (80% ACN, 0.4% TFA) and centrifuged at 3000 rpm for 2 min, followed by equilibrating in 200 μL of buffer B (80% ACN, 1% TFA, 0.3 mM lactic acid). Then the samples dissolved in 200 μL of buffer B were loaded onto the tip and centrifuged at 2000 rpm for 20 min. The flow-through was put back into the spin tip and centrifuged again. The tip was rinsed with 200 μL of buffer B, followed by 200 μL of buffer A three times. Finally, the peptides were eluted with 50 μL of 5% ammonium hydroxide and 50 μL of 5% pyrrolidine sequentially. The eluates were combined, desalted with a C18 micro spin column, and lyophilized to dryness for nanoRPLC–MS/MS analysis.

### CK2α1 Kinase Assay

Recombinant human CK2α1 kinase was purchased from Promega (stocked in 20 mM Tris-HCl (pH 8.0), 0.4 M urea, and 10% glycerol). Fifty ng of CK2α1 was incubated with recombinant human OGA (a final concentration of ∼100 ng/μL) in reaction buffer (25 mM Tris-HCl (pH 7.5), 10 mM MgCl_2_, 5 mM β-glycerophosphate, 2 mM DTT, and 0.1 mM Na_3_VO_4_) at 30 °C. Reaction was started by the addition of 1 mM AΤP for 30 min at 30 °C and was ended with the addition of 5 mM EDTA. Reaction without ATP served as a negative control.

### OGA Immunoprecipitation

OGA antibody (MGEA5 Antibody, Cat: A304–345A) was first immobilized on the AminoLink Plus beads (Thermo) according to the manufacturer’s instructions. One mg of cell lysates in the lysis buffer (with appropriate inhibitors) was incubated with Ab-conjugated beads for 12 h at 4 °C by gentle rotation. Then the beads were washed with lysis buffer three times, with proteins on the beads eluted by heating in 35 μL of SDS buffer (5% SDS in 50 mM TEABC, 20 mM DTT) for 10 min at 50 °C. Extracted proteins were digested with S-Trap processing as described in the Supporting Information.

### NanoRPLC–MS/MS Analysis

Peptides were analyzed with a nanoAcquity UPLC system (Waters) coupled with an Orbitrap Fusion Lumos mass spectrometer (Thermo Fisher) in either data-independent acquisition (DIA) mode (for proteomics and phosphoproteomics) or data-dependent acquisition (DDA) mode (for samples from immunoprecipitation and in vitro assays). In brief, samples were resuspended in 0.1% formic acid (FA) solution and loaded onto a C18 Trap column (Waters Acquity UPLC M-Class Trap, Symmetry C18, 100 Å, 5 μm, 180 μm × 20 mm) at 10 μL/min for 4 min. Peptides were then separated with an analytical column (Waters Acquity UPLC M-Class, peptide BEH C18 column, 300 Å, 1.7 μm, 75 μm × 250 mm), which was temperature controlled at 45 °C. The flow rate was set as 400 nL/min. A 150 min gradient of buffer A (2% ACN, 0.1% FA) and buffer B (0.1% FA in ACN) was used for separation: 1% buffer B at 0 min, 5% buffer B at 1 min, 22% buffer B at 105 min, 36% buffer B at 125 min, 50% buffer B at 130 min, 90% buffer B at 135 min, 90% buffer B at 145 min, 1% buffer B at 145.1 min, and 1% buffer B at 150 min. The MS data were acquired by an Orbitrap Fusion Lumos mass spectrometer using an ion spray voltage of 2.5 kV and an ion transfer temperature of 275 °C. Mass spectra were recorded with Xcalibur 4.0. Advanced peak determination was on for MS analyses.

DDA parameters were set as follows: detector type, Orbitrap; mass range, 375–1500 *m*/*z*; orbitrap resolution, 120,000; scan range, 375–1500 *m*/*z*; RF lens, 30%; AGC target, standard; maximum injection time mode, auto; microscans, 1; charge state, 2–7; exclusion duration, 40 s; and cycle time, 3 s. MS/MS parameters were set as follows: isolation mode, quadrupole; isolation window, 1.6 *m*/*z*; HCD normalized collision energy, 30%; detector type, Orbitrap; resolution, 30 000; and normalized AGC target, 200%.

The DIA-MS/MS scan was performed in the HCD mode with the methods consisting of one MS1 scan and 33 MS2 scans of variable isolated windows, with 1 *m*/*z* overlap between windows. On average, 4–5 data points per peak were achieved in the DIA measurements of 2.5 h in the present study. The MS1 scan range was 350–1650 *m*/*z*, and the MS1 resolution was 120,000 at *m*/*z* 200. The MS1 full scan AGC target value was set to be 4 × 10^5^, and the maximum injection time was 100 ms. The MS2 resolution was set to 30,000 at *m*/*z* 200 with the MS2 scan range 200–1800 *m*/*z*, and the normalized HCD collision energy was 30%. The MS2 AGC was set to be 5 × 10^4^, and the maximum injection time was 50 ms. The default peptide charge state was set to 2. Both MS1 and MS2 spectra were recorded in profile mode using positive polarity.

### Data Analysis

DDA raw files were processed in Proteome Discoverer (Thermo Fisher Scientific, version 2.4) with a Sequest HT database search engine. The human OGA sequence (UniProt accession: O60502) retrieved from the UniProt was used as a database for analysis of DDA data for samples from immunoprecipitation and in vitro assays. The database searching parameters were set as follows: full tryptic digestion and allowed up to two missed cleavages, the precursor mass tolerance was set at 10 ppm, whereas the fragment-mass tolerance was set at 0.02 Da. Carbamidomethylation of cysteines (+57.0215 Da) was set as a fixed modification, and variable modifications of methionine oxidation (+15.9949 Da) and acetyl (N-terminus, +42.011 Da) were allowed. The false discovery rate (FDR) was determined by using a target-decoy search strategy. The decoy-sequence database contains each sequence in reverse orientation, enabling FDR estimation. On the peptide level, the corresponding FDR was <1%.

DIA data files were processed with Spectronaut (Biognosys, v18) using standard settings with slight modifications for the quantification of protein abundances and phosphorylation levels.^29^ In brief, dynamic retention time prediction with local regression calibration was selected. Interference correction on MS and MS^2^ levels was enabled. The *Q*-value cutoff was set to 1% at peptide precursor and protein levels using scrambled decoy generation and dynamic size at 0.1 fraction of library size. MS^2^-based quantification by area was used, enabling local cross-run normalization. At a specific modification site, quantitative site collapse of parent peptides carrying phosphorylation was performed. If the parent peptides carried more phosphorylated sites, a separate collapse was performed according to the modification multiplicity. All of the phosphosites with a localization probability of ≥0.75 were filtered by a *p*-value of 0.05 and an absolute log_2_ ratio of 1. In the Spectronaut quantity report, values labeled as “Filtered” indicate cases where a feature was detected and evaluated, but the quantitative value did not pass the internal quality-control criteria for that specific run.

Other experimental procedures are shown in the Supporting Information.

## Results and Discussion

### Quantitative Proteomics and Phosphoproteomics of PANC-1 Cells upon Disruption of O-GlcNAc Cycling

To investigate the crosstalk between O-GlcNAcylation and phosphorylation, we performed systematic quantification of the proteome and phosphoproteome of PANC-1 cells upon acute treatment with OSMI-1 (a specific inhibitor of OGT) and TMG (a specific inhibitor of OGA), and without treatment (DMSO group). Figure 1A shows an overview of the experimental design. In brief, proteins extracted from cells upon each treatment were digested with trypsin. A small portion (1%) of the digests was subjected to direct nanoRPLC–MS/MS analysis in label-free data-independent acquisition (DIA) mode for total protein quantitation. The remaining peptides (99%) were subjected to TiO_2_ bead-based enrichment, with enriched phosphopeptides analyzed on the same nanoRPLC–MS/MS system in label-free DIA mode for quantification of phosphopeptides and phosphosites.

**Figure 1:**
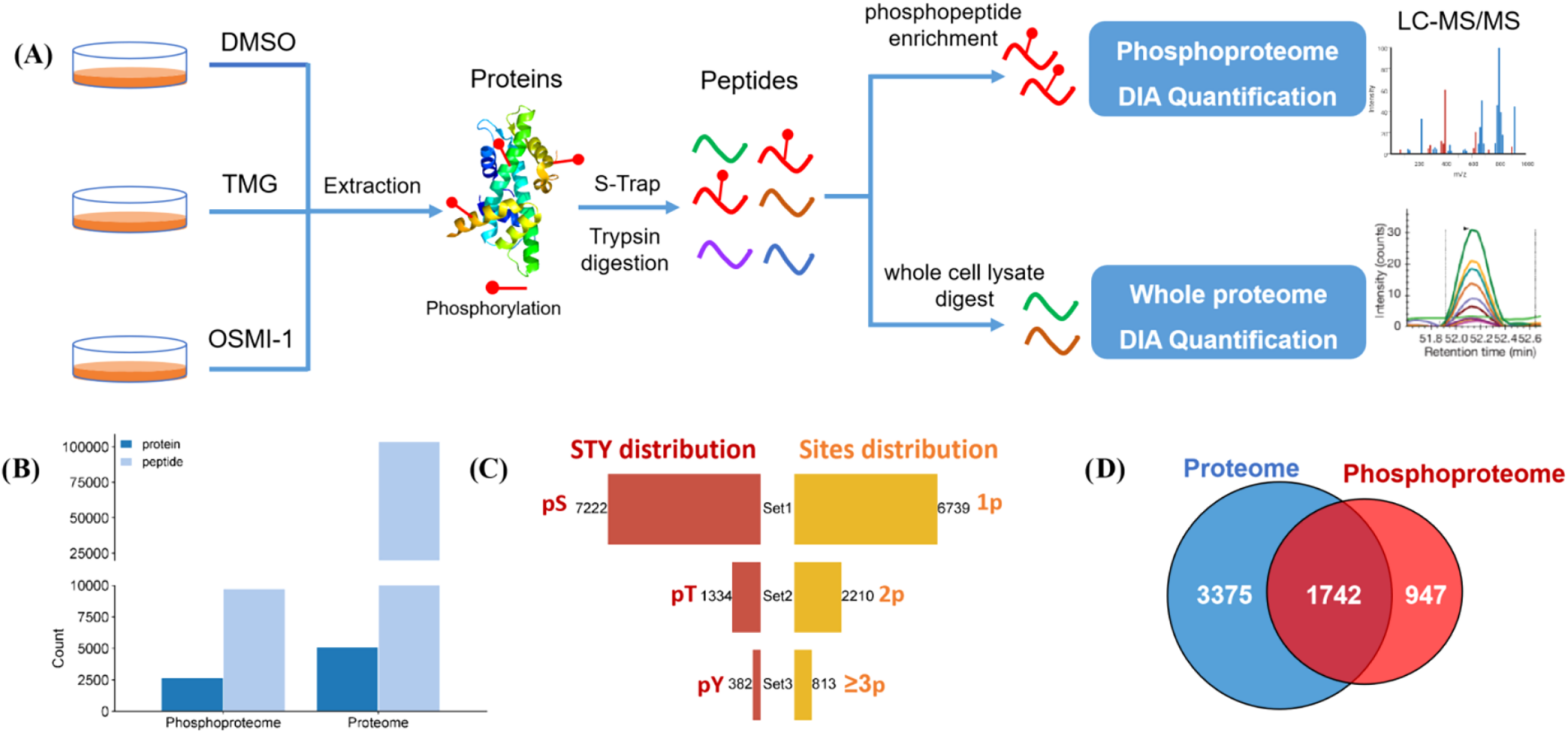
(A) Experimental design for proteomics and phosphoproteomics of PANC-1 cells upon treatment with DMSO, TMG, and OSMI-1 (*n* = 3). (B) Total numbers of (phospho)­peptides and (phospho)­proteins identified from proteomics and phosphoproteomics. (C) Distribution of mono- (1p), double- (2p), and triple- (3p) phosphorylated peptides and phosphorylation on serine (S), threonine (T), and tyrosine (Y). (D) Venn diagram of the number of total proteins and phosphoproteins identified.

In total, 94,851 peptides corresponding to 5117 proteins were identified and quantified across samples in the proteomics study. Phosphoproteomics yielded a total of 9762 phosphopeptides corresponding to 2689 phosphoproteins (Figure 1B). As shown in Figure 1C, a total of 8938 phosphosites were identified, including 7222 phosphoserine sites (80.8%), 1,334 phosphothreonine sites (14.9%), and 382 phosphotyrosine sites (4.3%). The distribution of phosphopeptides was 6739 mono- (69.0%), 2210 double- (22.6%), and 813 triple-phosphorylated peptides (8.3%), which is comparable to that reported in previous work.^30,31^ Of note, an additional 947 proteins were identified from phosphoproteomics analysis (Figure 1D). The substantial overlap of proteins identified from both the whole proteome and the phosphoproteome allows us to further analyze the dynamic changes in phosphorylation at both the protein and site-specific levels. Collectively, the comprehensive data sets enabled a detailed examination of how phosphorylation events are modulated across different conditions, offering deep insights into the functional implications of these modifications.

First, total protein quantification was performed, with the corresponding volcano plots presented in Figures 2A, 2B. Among the 5117 proteins quantified, only 16 proteins were upregulated, and 36 were downregulated following TMG treatment. Conversely, after OSMI-1 treatment, only 19 proteins were upregulated, and 24 were downregulated. Further analysis revealed no significant differences in overall protein quantification ratios between the OSMI-1/DMSO and TMG/DMSO groups (Figure 2C, Table S1). Taken together, these data suggest that an acute treatment (4 h) with OSMI-1/TMG does not lead to overall changes to the proteome, with levels of >99% of total proteins unaffected.

**Figure 2:**
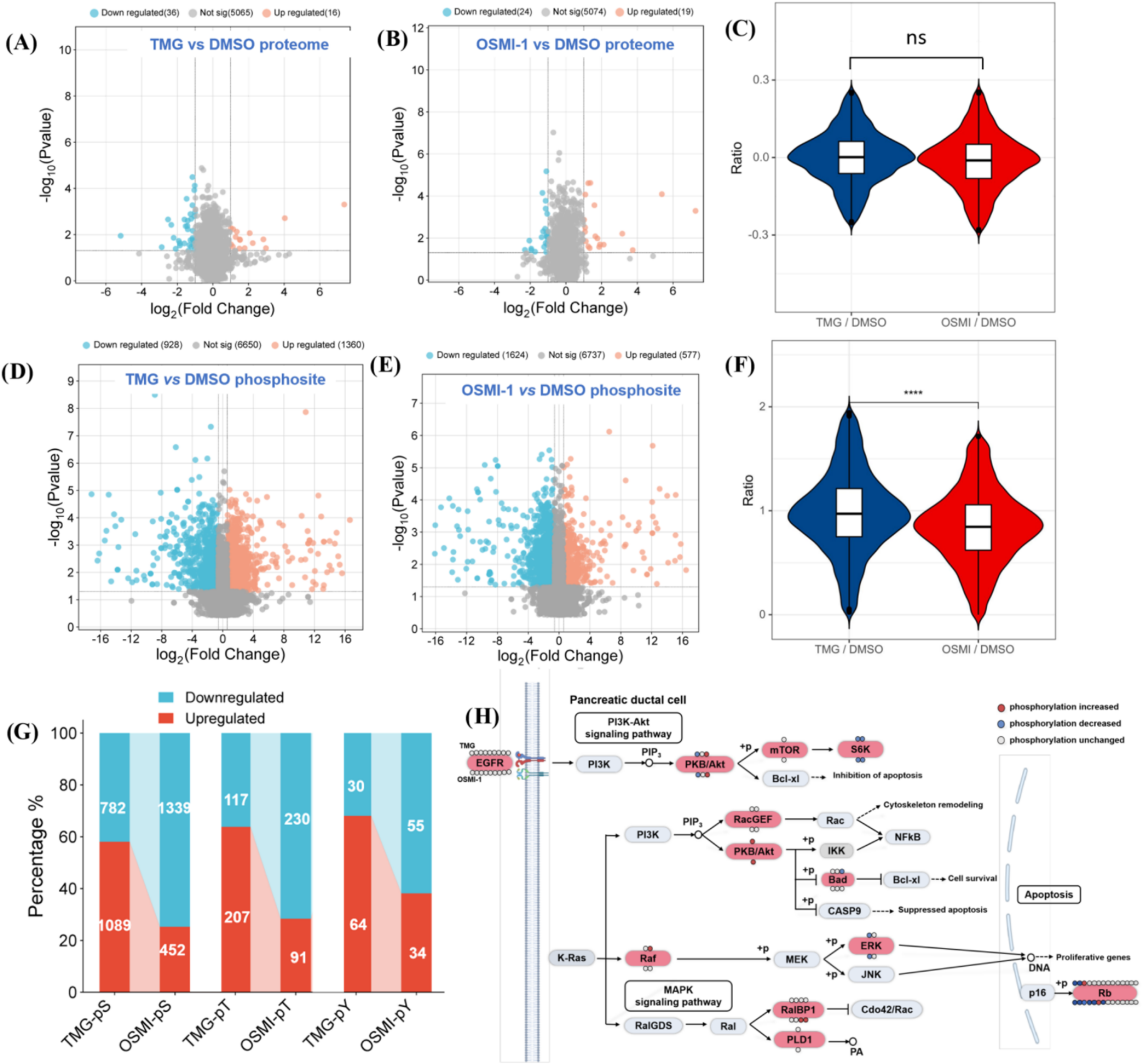
Quantitative analysis of proteomics and phosphoproteomics in PANC-1 cells upon different treatments. Volcano plots of the proteins in (A) the TMG group and (B) the OSMI group compared with the DMSO group. (C) Overall proteome changes between the TMG/DMSO and OSMI/DMSO groups (ns: not significant, *p* > 0.05). Volcano plots of phosphosites in (D) the TMG group and (E) the OSMI group compared with the DMSO group. (F) Violin plot of ratio distribution of quantified phosphosites between the OSMI/DMSO and TMG/DMSO groups (****: *p* < 0.0001). (G) Distribution of dysregulated phosphorylation on S, T, and Y for the OSMI/DMSO and TMG/DMSO groups. (H) KEGG analysis of the pancreatic cancer-related pathway.

Quantitative and site-specific phosphoproteomics was then performed for cells after treatment with the two O-GlcNAc cycling inhibitors. An acute treatment of 4 h with TMG led to significant changes in 2289 phosphosites on 1225 proteins (Figure 2D, Table S2). Among them, phosphorylation on 1360 sites (59%) was increased, and that on 929 sites (41%) was decreased. When treated by OSMI-1, 2201 phosphorylated sites on 1199 proteins were significantly altered (Figure 2E). Among these sites, the up- and downregulated site numbers were 577 (26%) and 1624 (74%), respectively. The ratio distribution of quantified phosphosites between the OSMI/DMSO and TMG/DMSO groups reveals that the overall phosphorylation level in the TMG treatment group is significantly higher than that in the OSMI-1 treatment group (Figure 2F). Interestingly, there was no overlap between the significantly changed proteins and phosphoproteins, indicating that the inhibition of OGA and OGT affects phosphorylation variation at the site level rather than at the protein level. The distribution of regulated phosphorylated residues shows that the majority of significantly changed phosphosites under TMG and OSMI-1 treatment are S and T residues, constituting approximately 82% and 14%, respectively (Figure 2G). When treating with TMG, the proportion of upregulated phosphoserine and phosphothreonine was ∼1.5–2 fold higher than that of downregulated ones. However, most phosphoserine and phosphothreonine sites were downregulated in the OSMI-1-treated group. These data demonstrate that increased O-GlcNAcylation in the proteome promotes phosphorylation on the majority of modification sites and decreased O-GlcNAcylation reduces phosphorylation on >70% of modification sites on proteins. Notably, approximately 90 phosphotyrosine sites were also affected, with the most being upregulated in the TMG groups and downregulated in the OSMI-1 group. This is potentially linked to the changes in phosphorylation of protein tyrosine phosphatases, which will be analyzed in the following section. Very interestingly, phosphorylation on a tyrosine site Y2516 in CHD8 (Chromodomain-helicase-DNA-binding protein 8, Q9HCK8), a chromatin remodeling factor,^32^ was upregulated in both the TMG and OSMI-1 treatment groups. Given that this site was previously identified as O-GlcNAcylated in PANC-1 cells in our prior work,^4^ site-specific crosstalk between phosphorylation and O-GlcNAcylation on Y2516 of CHD8 and its regulatory effects are worth exploring.

KEGG pathway analysis illustrates the assortment of significantly regulated genes and molecular interactions implicated in the pathogenesis of pancreatic cancer (Figure 2H). For example, this analysis identified 17 proteins undergoing phosphorylation within the PI3K/Akt pathway, an aberrantly activated pathway frequently found in pancreatic cancer,^33,34^ with phosphorylation on 10 proteins found to be dysregulated. Moreover, our phosphoproteomics identified seven phosphorylated proteins (including RAF1, ARAF, BRAF, MAPK3, and MAPK1) with 32 phosphosites of the MAPK signaling pathway, a critical driver of pancreatic cancer.^35,36^ Remarkably, 9 phosphosites of the proteins from this pathway were dysregulated when cells were treated with TMG or OSMI-1. Of note, a total of 14 phosphosites were identified from RB1 (retinoblastoma-associated protein 1, P06400), a tumor suppressor protein, whose hyperphosphorylation drives the proliferation of PDAC.^37,38^ Upon TMG treatment, we observed one upregulated phosphosite (S798) and two downregulated phosphosites (S608 and S612) on RB1. Concomitantly, OSMI-1 treatment reduced phosphorylation levels on five modification sites (S612, S608, S807, and S811, T826) on RB1, simultaneously with only one phosphosite upregulated (S794). More specifically, two clusters of phosphorylation regions, namely S608/S612 and T807/T811, have been documented to modulate the intramolecular interaction with the transcription factor E2F binding surface.^37^ OSMI-1 treatment decreased phosphorylation levels both at S608/S612 and T807/T811 (analogous to hyperphosphorylation, which drives the proliferation of PDAC), whereas TMG treatment only significantly decreased the phosphorylation levels at S608/S612. Moreover, RALBP1 (RalA-binding protein 1, Q15311) has been reported to promote cytoskeletal rearrangement, facilitating cancer cell migration and invasion.^39^ Our analysis quantified four phosphorylation sites, and treatment with OSMI-1 led to increased phosphorylation at T34 and T36, while no significant dysregulation was observed following TMG treatment. Taken together, our phosphoproteomics data suggest that TMG treatment may exacerbate phosphorylation on many proteins in key signal transduction pathways in pancreatic cancer cells, while OSMI-1 effectively decreases it. These results further support the notion that inhibiting O-GlcNAcylation, e.g., by using OGT-specific inhibitors such as OSMI-1, serves as a promising strategy for cancer treatment (including pancreatic cancer).^11,26–28,40–46^

To get a deeper and more comprehensive look at the phosphoproteomics data, we classified phosphoproteins with dysregulated phosphosites identified upon OSMI-1 or TMG treatment into five major clusters (Figure 3, Table S3). These clusters included sites with opposite phosphorylation changes in the TMG and OSMI-1 groups (clusters 2 and 4), sites with increased or decreased phosphorylation in both the TMG and OSMI-1 groups (clusters 1 and 5), and sites showing a consistent downregulation trend (cluster 3). Gene ontology (GO) analysis of the corresponding proteins revealed substantial functional differences among the clusters. For example, proteins with phosphosites downregulated in the OSMI-1 group but upregulated in the TMG group (cluster 2) were highly enriched in the processes of negative regulation of organelle organization and DNA repair. Those with phosphosites upregulated in the OSMI-1 group and downregulated in the TMG group (cluster 4) were mainly associated with biological processes, including organelle localization, cellular response to epidermal growth factor stimulus, and regulation of translation. Interestingly, some proteins showed markedly upregulated phosphosites in both OSMI-1 and TMG treatments but with a greater increase in the TMG group (cluster 1). These proteins were primarily enriched in biological processes such as chromatin remodeling, actin filament-based processes, regulation of cytoskeleton organization, and RNA splicing. In contrast, proteins with phosphosites that were downregulated in both OSMI-1 and TMG treatments but with a more pronounced decrease in the TMG group (cluster 5) were enriched in mRNA metabolic processes, positive regulation of protein localization, and ribonucleoprotein complex biogenesis. Finally, proteins with consistently downregulated phosphosites (cluster 3) were mainly enriched in biological processes such as regulation of autophagy and translational initiation. As for the cellular component (CC) and molecular function (MF) analyses (Figure S1), the CC enrichment highlights various cellular structures and regions with significant associations to the clusters, including the centrosome, ribonucleoprotein granule, and cell–cell junction. Each cluster shows distinct enrichment patterns, with cluster 1 and cluster 2 showing a prominent association with ribonucleoprotein granules, while cluster 3 is enriched in the chromosomal region and cell–substrate junction. The MF enrichment reveals the molecular functions associated with the proteins in each cluster, with key terms like transcription factor binding, GTPase regulator activity, and kinase binding being notably enriched in clusters 1 and 2. Cluster 3 stands out for its enrichment in microtubule and actin filament binding, while other clusters show varying levels of association with functions like mRNA binding and protein kinase activity. Collectively, these data suggest that disrupted O-GlcNAcylation is involved in virtually all biological processes, partially through the modulation of phosphorylation on diverse classes of cellular proteins.

**Figure 3:**
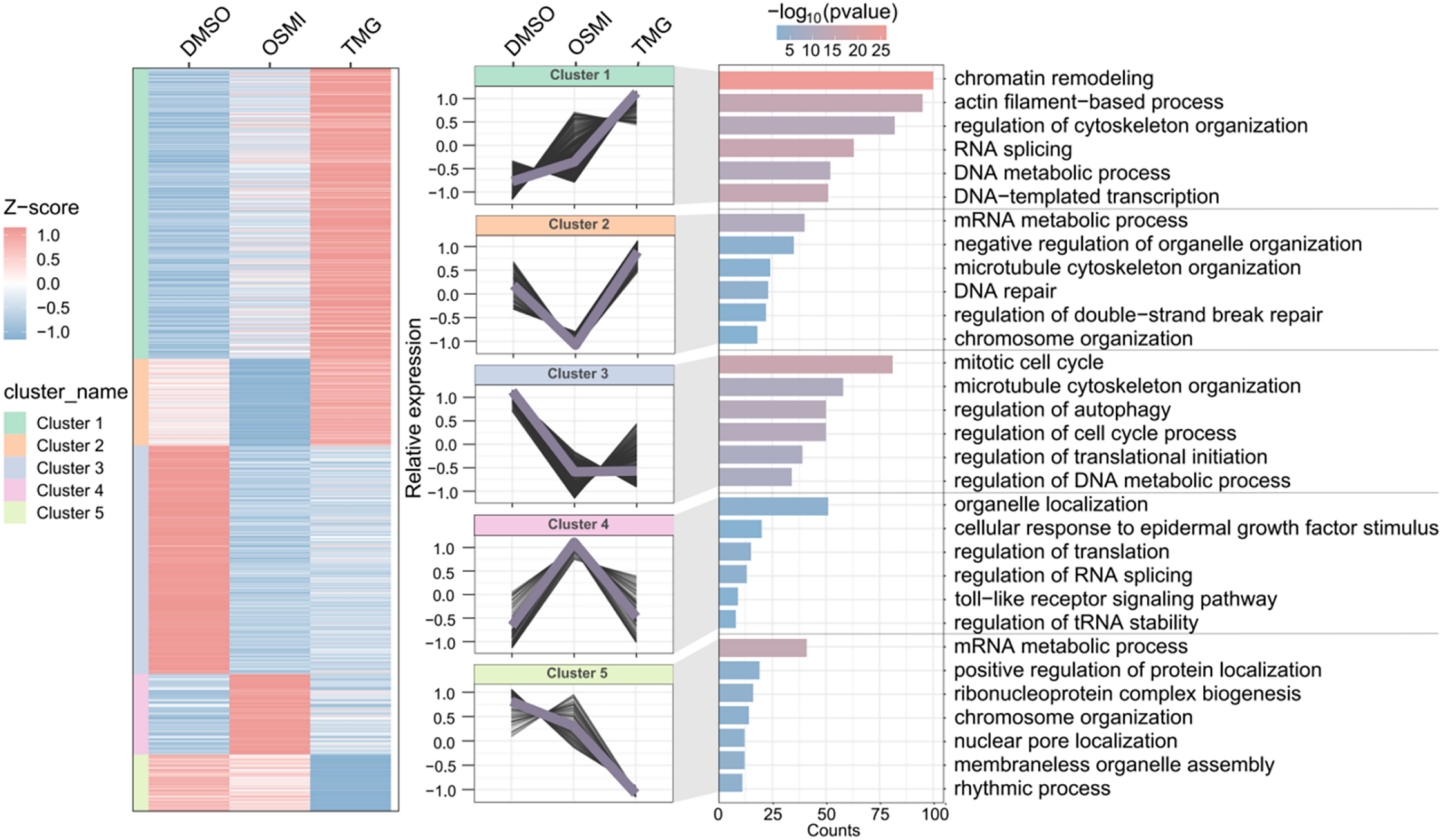
Distribution of dysregulated phosphosites identified upon OSMI-1 or TMG treatment, visualized by a heatmap of Z-scored mean protein-psite intensities from three replicates (left). Phosphoproteins with dysregulated phosphosites were grouped into five clusters using hierarchical clustering (Euclidean distance, complete linkage), with cluster assignments indicated by the color bar (middle). Biological process (BP) enrichment analysis was performed on proteins corresponding to each cluster (right). The color gradient indicates the significance of enrichment, with darker shades representing higher significance levels.

Moreover, we analyzed the phosphorylation patterns using the motif-x algorithm of MoMo in MEME Suite 5.4.1,^47^ with parameters as follows: motifs of at least 20 occurrences; 15 residues wide; shuffled foreground peptides as background; score threshold of 0.000001, adjusted *p*-value <0.05. As shown in Table S4, 72 motifs were enriched from 15 residues flanking regions of all phosphorylated sites that were identified in DMSO-, OSMI-, and TMG-treated samples, including “xxxxxxx_S_Pxxxxxx” with the lowest adjusted p-value of 2.00e^–30^. These motifs were subtracted from those discovered based on the significantly changed phosphorylated sites sensitive to OSMI or TMG treatments. Therefore, 26 and 28 motifs were enriched from flanking regions of phosphorylated sites that were affected by OSMI and TMG, respectively. The first three motifs with the lowest adjusted p-values that were discovered under OSMI conditions are “xxxxxxL_S_Pxxxxxx” (9.10e^–20^), “xxxxxxx_S_PxKxxxx” (1.40e^–17^), and “Rxxxxxx_S_Pxxxxxx” (2.80e^–16^). The first three motifs with the lowest adjusted p-values that were discovered under TMG conditions are “xxRxRxx_S_xxxxxxx” (5.90e^–20^), “xxxxxxx_S_PxxxxTx” (4.60e^–19^), and “xxxxxxx_S_PxKxxxx” (1.90e^–18^).

Last but not least, we wondered how many of the phosphoproteome changes might be direct events (e.g., a site that can undergo phosphorylation and O-GlcNAcylation). Previously, we performed deep O-GlcNAc proteomic profiling of PANC-1 cells with the same TMG treatment, with 2831 O-GlcNAc sites identified.^4^ By comparing that data set with phosphoproteomics data sets obtained in this study, we found that only 67 sites can be either phosphorylated or O-GlcNAcylated (Table S5). Among them, only about a dozen sites showed significant phosphorylation changes upon TMG or OSMI-1 treatment. In this sense, these results indicate that the majority, if not all, of the phosphorylation changes are indirect events rather than direct events (e.g., resulting from site-specific reciprocal competition between phosphorylation and O-GlcNAcylation). Although it is somewhat unexpected, this observation aligns with previous proteomics studies, which suggest sporadic overlaps between phosphorylation and O-GlcNAcylation sites.^48,49^ However, it is still likely that O-GlcNAcylation on some sites may modulate phosphorylation on adjacent residues and vice versa.

### Alteration of the Kinome and Phosphatome in PANC-1 Cells upon Disruption of O-GlcNAc Cycling

Kinases and phosphatases intricately regulate the phosphorylation status of their target proteins, achieving precise control and specificity within signaling pathways through multiple regulatory feedback loops.^1,2^ Prompted by our observation that only a small percentage of residues undergo phosphorylation and O-GlcNAcylation, we reasoned that the vast majority, if not all, of the phosphorylation changes might probably be indirect events (secondary/tertiary events), e.g., due to altered activity of kinases and phosphatases, which themselves are differentially phosphorylated. To that end, we investigated the phosphorylation changes in kinases and phosphatases upon OGA or OGT inhibition.

By referencing the human kinome database (http://www.kinhub.org/kinmap/), we identified 144 protein kinases in our data set, 96 of which were dysregulated following treatment with TMG or OSMI-1 (Figure 4A, Table S6). The protein kinases identified were distributed fairly evenly across the dendrogram of the human kinome. Further analysis revealed significant changes at 119 sites from 71 kinases upon OSMI-1 treatment and 124 sites from 80 kinases following TMG treatment. These kinases exhibited different distributions across categories of the kinase superfamily (Figure 4B). The substantial overlap of affected kinases and corresponding phosphosites between the two treatments suggests a conserved pattern of protein distribution across different kinase groups, as depicted in Figure 4C,4D. By specifically examining the distribution of modified sites within kinase groups, we found that OSMI-1 treatment leads to fewer upregulated and more downregulated sites compared to TMG, particularly within the CMGC group (Figure 4E,4F). These findings underscore the nuanced differential impacts of OSMI-1 and TMG on kinase regulation, highlighting their distinct roles in the cellular signaling pathways.

**Figure 4:**
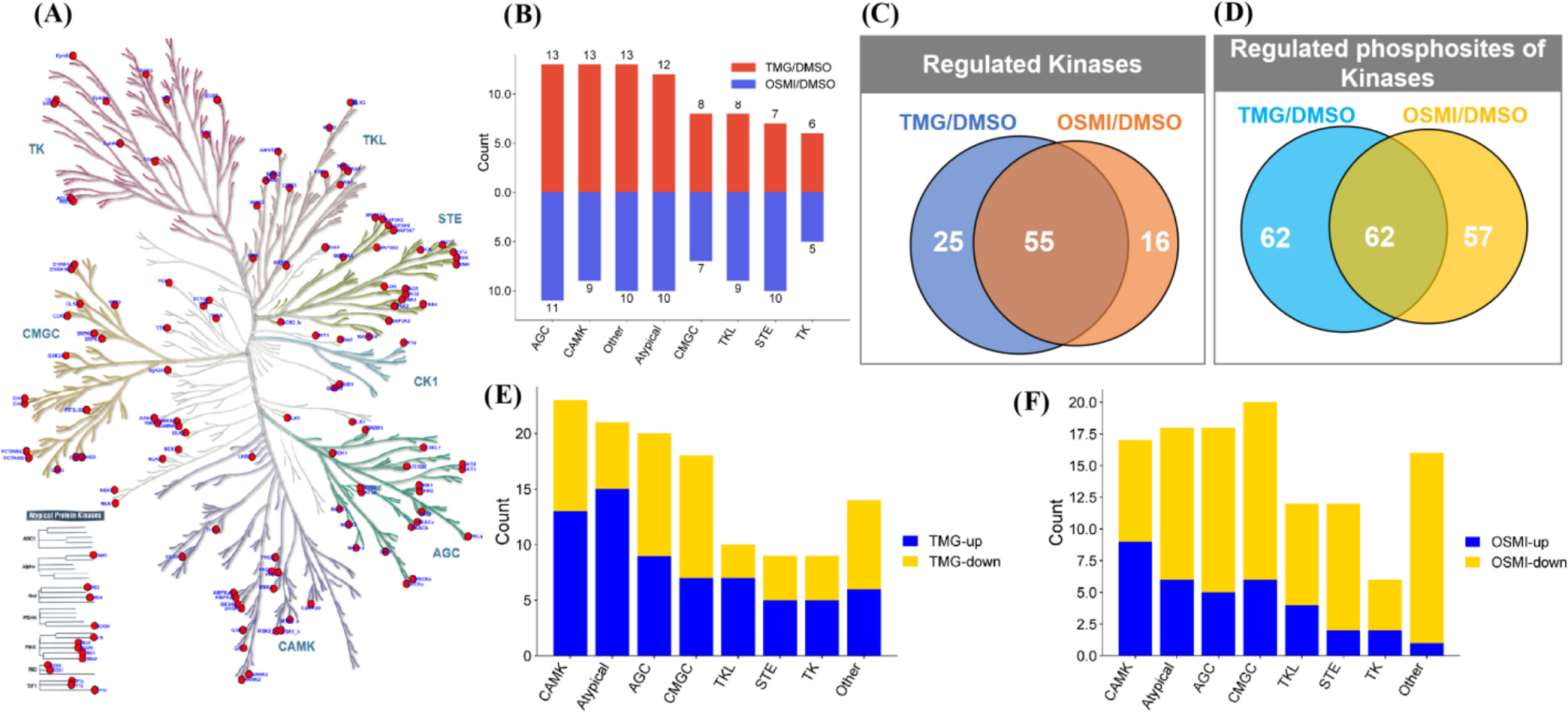
Kinase phosphorylation analysis. (A) Kinome tree analysis of the phosphorylation kinases identified in this work (red balls in the dendrogram indicate marked features, the blue text represents kinase genes). (B) Distribution of changed kinases across the superfamily after treatment. (C) Changes in the number of phosphorylated kinases upon treatment with inhibitors. (D) Changes in the number of phosphorylated sites on kinases after treatment with inhibitors. (E) Distribution of altered phosphorylation sites across different kinase families upon TMG treatment. (F) Distribution of altered phosphorylation sites across different kinase families upon OSMI-1 treatment.

By mapping to the human phosphatase database (http://www.phosphatome.net/), 102 phosphatases with 186 phosphosites were identified (Table S7). As shown in Figure 5A, seven types of phosphatase families were identified, and 64% of which were from the CC1-fold class, which is also reported to be the largest group of phosphatases comprising 106 members.^6^ We found that 23 sites from 17 phosphatases and 37 sites from 21 phosphatases were significantly changed by OSMI and TMG treatment, respectively (Figure 5B and 5C). A large overlap of phosphatases bearing changed sites was obtained, leading to a similar profile of protein distribution among different phosphatase superfamilies. For the site distribution among different superfamilies, TMG treatment induced prominently increased phosphorylation across phosphatase superfamilies (except in PPPL), while OSMI treatment resulted in overwhelmingly downregulated phosphorylation, particularly in CC1 (Figure 5D,5E). Protein tyrosine phosphorylation, meticulously balanced by the interplay of protein tyrosine kinases (PTKs) and phosphatases (PTPs), intricately regulates numerous cellular functions, including metabolism, cell growth, differentiation, migration, and apoptosis.^50–52^ When this equilibrium is disrupted, it can lead to a range of abnormalities, such as cancer, metabolic disorders, and immunological conditions.^53,54^ Consequently, PTPs have emerged as promising targets for therapeutic intervention. In this study, a cohort of PTPs, including PTN2, PTN12, and PTN14, was identified with 22 dysregulated phosphorylation sites. For instance, protein tyrosine phosphatase nonreceptor 12 (PTN12, Q05209), also known as PTP–PEST, predominantly localized in the cytoplasm, orchestrates various cellular processes through its phosphatase activity.^55^ In pancreatic cancer, PTPN12 negatively regulates key receptor tyrosine kinases such as ERBB2 (HER2), which are often overactive in cancer cells.^56,57^ By dephosphorylating these receptors, PTPN12 can limit the downstream signaling that drives tumor growth and metastasis. Loss or reduced expression of PTPN12 is associated with poor outcomes in several cancers, as it leads to unchecked receptor signaling and promotes tumor progression. During TMG treatment, 3 phosphorylation sites (S603, S606, and S608) of PTN12 were upregulated, with one site downregulated (S507). Conversely, upon treatment with OSMI-1, only the phosphorylation sites of S507 exhibited downregulation. These findings collectively suggest that the inhibition of OGA and OGT exerts distinct effects on protein tyrosine phosphatases, thereby regulating tyrosine phosphorylation dynamics differentially.

**Figure 5:**
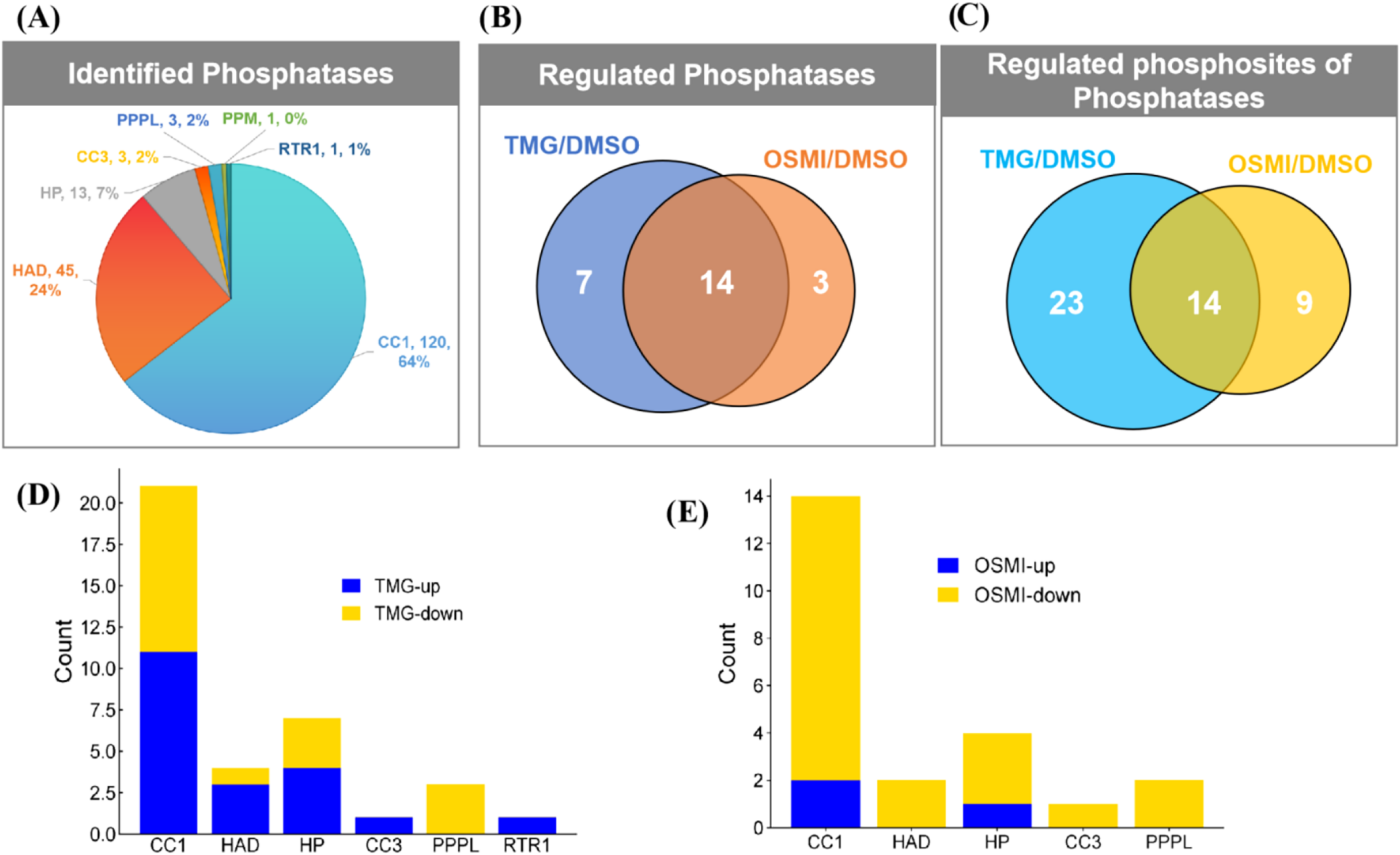
Phosphatase phosphorylation analysis. (A) Distribution of phosphorylated phosphatases. (B) Changes in the number of phosphorylated phosphatases after treatment with inhibitors. (C) Changes in the number of phosphosites on phosphatases after treatment with inhibitors. (D) Distribution of altered phosphosites across different phosphatase groups upon TMG treatment. (E) Distribution of altered phosphosites across different phosphatase groups upon OSMI-1 treatment.

We employed STRING and Cytoscape tools to visualize the interactions among differentially expressed kinases (96 proteins) and phosphatases (24 proteins) with OGA and OGT (Figure S2). The analysis clearly demonstrates that the OGA and OGT proteins are intricately linked with the kinases and phosphatases in the network. It reveals key insights into how these enzymes may modulate signaling networks, potentially impacting cellular functions and disease mechanisms. For example, cyclin-dependent kinase 13 (CDK13, Q14004), which is believed to phosphorylate RNA polymerase II and is involved in transcriptional activation, also catalyzes other protein substrates and contributes to tumorigenesis.^58^ Ten phosphorylated sites on CDK13 were identified, and six sites were dysregulated following treatment with TMG (five phosphosites S525, T531, S328, T871, and S325 upregulated) and OSMI-1 (three phosphosites S525, T531, and S328 upregulated; S1153 downregulated). Additionally, two key components of the translation machinery, 4E-BP1 and eIF4B, which are CDK13 substrates, were identified. CDK13 directly phosphorylates eIF4B (P23588) at S422,^59^ a site which was found to be upregulated after treatment with both TMG and OSMI-1. Three other phosphorylated sites (S93, T205, and S283) of eIF4B were identified as downregulated in both the TMG- and OSMI-1-treated samples. Additionally, phosphosites of S207 on eIF4B were found to be upregulated in the TMG group.

### Alteration of OGT and OGA in PANC-1 Cells upon Disruption of O-GlcNAc Cycling

We next examined the potential changes of OGT and OGA, the two enzymes controlling O-GlcNAc cycling. From quantitative proteomics, we did not find significant protein-level changes of OGT and OGA, demonstrating that acute treatment with OGT/OGA inhibitors does not induce their protein-level changes. Although previous reports showed that OGT is a substrate of several kinases,^60^ OGT was not among the 2689 phosphoproteins identified in the current data sets. In contrast, OGA was found to be phosphorylated on several sites, including S364, T370, Y374, and S522 (Table S2). Very strikingly, up to 18-fold increased phosphorylation on S522, a site located in the helical bundle of OGA, was observed after the TMG treatment. Interestingly, the level of phosphorylation on S364 was decreased by 30%. These results indicate that OGA, an indispensable O-GlcNAc cycling component, is potentially regulated by phosphorylation, which is responsive when the intracellular O-GlcNAcylation homeostasis is perturbed. The altered phosphorylation on the O-GlcNAc cycling enzymes may be another key contributor to the intricate crosstalk between phosphorylation and O-GlcNAcylation.

### CK2α-Mediated Phosphorylation of OGA at Serine 364

Given that S364, a site localized in the catalytic domain of OGA, was phosphorylated and downregulated under TMG treatment, we next explored its potential kinases. Using the Scansite4 analysis software,^61^ we performed kinase prediction for the phosphorylation of S364 on the OGA site. Casein kinase 2 α (CK2α), a serine-threonine protein kinase that targets many critical regulators of cellular growth,^62–64^ was ranked as a strong candidate. Intriguingly, CK2α, the catalytic subunit of the ubiquitously expressed and constitutively active kinase CK2, has been reported to be modified by O-GlcNAc, and this modification has critical effects on the phosphorylation of its substrates.^65–67^

To test whether CK2α is the kinase that can phosphorylate S364 of OGA, we treated PANC-1 cells with CX-4945 (Silmitasertib), a first-in-class, orally available, and highly selective inhibitor of CK2 for the treatment of various solid tumors.^66–69^ OGA was immunoprecipitated from cells, with the eluate digested and then analyzed with nanoHPLC-MS/MS to identify and quantify the phosphorylation of OGA. Cells treated with DMSO and TMG were used as controls and processed in parallel. The phosphorylation status of OGA was compared among groups. As shown in Figure 6A and 6B, phosphorylation on S364 was robustly quantified. The representative mass spectrum of the phosphorylated peptide (LENEGSpDEDIETDVLYSPQMALK) is shown in Figure 6C. More importantly, significantly decreased phosphorylation on S364 of OGA was observed after silmitasertib treatment, which was even more pronounced than that of TMG-treated cells. These results strongly suggest that phosphorylation at the S364 site on OGA is tightly regulated by CK2 activity. The observed decrease in phosphorylation on S364 upon CK2 inhibition with silmitasertib may suggest the direct involvement of CK2 in catalyzing this phosphorylation event. Additionally, the reduction in phosphorylation on S364 in response to OGA inhibition by TMG indicates a functional interplay or feedback mechanism between OGA enzymatic activity and its own phosphorylation status. The disrupted S364 phosphorylation by either the CK2 inhibitor or the OGA inhibitor underscores the complexity of post-translational regulation within this pathway and suggests that OGA activity and CK2-mediated phosphorylation are probably interdependent.

**Figure 6:**
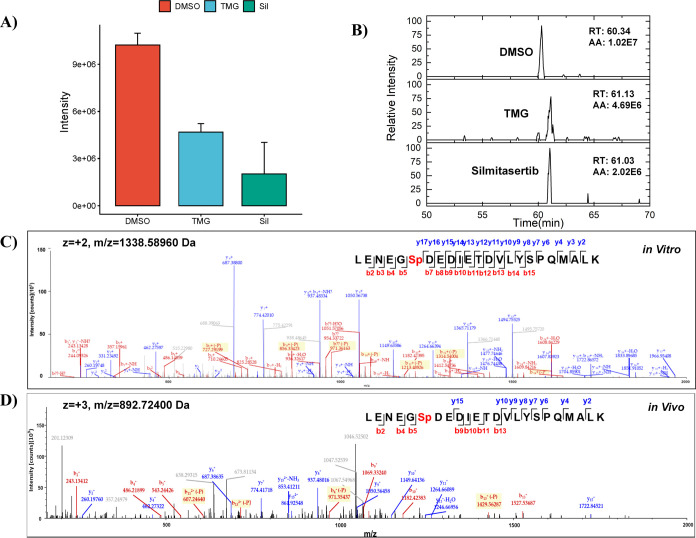
Phosphorylation at S364 of OGA following treatments with TMG and silmitasertib. (A) Intensity of phosphorylated S364 of OGA from PANC-1 cells treated with TMG, silmitasertib, or DMSO (*n* = 3). (B) Extracted ion chromatograms of the S364-containing phosphopeptide from three treatment groups. The mass spectrum of the S364-containing phosphopeptide identified from *in vitro* assays (C) and from cells (*in vivo*) (D).

To further confirm OGA S364 is a bona fide target of CK2α, we conducted *in vitro* kinase assays by incubating recombinant OGA and CK2α. In the absence of CK2α, no phosphorylation was detected on OGA. However, upon addition of CK2α, we observed robust and highly specific phosphorylation at S364 of OGA (Figure 6C), demonstrating that CK2α is the kinase responsible for this modification. The highly similar b and y ion patterns in the mass spectrum to those obtained from the *in vivo* experiments illustrate the accurate identification of the modification (Figure 6D).

Previous studies have shown that the CK2α itself is subjected to O-GlcNAcylation at S347, a modification mediated by OGT.^65–69^ Our findings here suggest that there is potentially a complex interplay between phosphorylation and O-GlcNAcylation: OGT catalyzes the O-GlcNAcylation of CK2α at Ser347, while CK2α phosphorylates OGA at S364. OGA, in turn, removes O-GlcNAc modifications from CK2α and other substrates. Notably, inhibition of either CK2α or OGA disrupts the phosphorylation of OGA at S364, underscoring the potential interdependence and dynamic regulation between these two PTMs. Although not addressed here, it is worthwhile to explore whether phosphorylation of OGA at S364 affects its functions (such as catalytic activity, stability, localization, or substrate engagement) in cells and whether there is a potential regulatory loop involving mutual modulation between phosphorylation and O-GlcNAcylation mediated by CK2α, OGT, and OGA.

## Conclusions

In summary, we conducted a systematic analysis of the crosstalk between O-GlcNAcylation and phosphorylation by quantifying (phospho)­proteome changes of the PANC-1 cell) treated with OSMI-1 (a specific inhibitor of OGT) or TMG (a specific inhibitor of OGA). We quantified 8938 phosphorylation sites on 2689 proteins, with 3527 phosphosites from 1628 proteins significantly altered by either the OSMI-1 or TMG treatment. An in-depth analysis of the kinome and phosphatome revealed extensive crosstalk between O-GlcNAcylation and phosphorylation, even after short-term perturbation with O-GlcNAc cycling inhibitors.

These results provide a rich data set that enhances our understanding of the interaction between protein phosphorylation and O-GlcNAcylation. For example, our data sets indicate that a huge majority of phosphorylation changes are probably indirect events (secondary/tertiary events; e.g., due to altered activity of kinases and phosphatases), rather than site-specific reciprocal competition between phosphorylation and O-GlcNAcylation. This observation awaits further validation by quantitative O-GlcNAc proteomics. Moreover, the revelation of a new kinase toward OGA phosphorylation (i.e., CK2α) is intriguing, while how it may regulate OGA’s function is worthy to explore.

Although the acute treatment (4 h) in PANC-1 cells is meaningful, longer treatment time points beyond the 4 h mark in this and other types of cells would provide valuable insights into the chronic effects upon prolonged O-GlcNAcylation disruption. Moreover, quantitative O-GlcNAc proteomics, which would offer critical views of the O-GlcNAcylated substrates (e.g., kinases and phosphatases), should be pursued in future studies. The integration of these strategies will greatly facilitate the investigation of the dynamic changes of cellular signaling networks, which will enable us to have a deeper understanding of the regulatory mechanisms and pathways involved in cellular processes and potentially identify key targets for therapeutic intervention.

## Supplementary Material





## Data Availability

The mass spectrometry proteomics data have been deposited to the ProteomeXchange Consortium (https://proteomecentral.proteomexchange.org) via the iProX partner repository with the data set identifier PXD070284.

## Notes

Table S1. List of quantified proteins of PANC-1 cells upon disruption of O-GlcNAc cycling with TMG or OSMI-1. Table S2. List of quantified phosphorylation sites of PANC-1 cells upon disruption of O-GlcNAc cycling with TMG or OSMI-1. Table S3. List of classified phosphoproteins with dysregulated phosphosites identified after OSMI-1 or TMG treatment, grouped into five major clusters. Table S4. List of phosphorylation patterns using the motif-x algorithm of MoMo. Table S5. List of overlapped modification sites. Table S6. List of kinases with site-specific phosphorylation changes after TMG or OSMI-1 treatment. Table S7. List of phosphatases with site-specific phosphorylation changes after TMG or OSMI-1 treatment (XLSX)
Figure S1. Cellular component (CC) and molecular function (MF) enrichment analyses were performed for proteins corresponding to the five clusters. Figure S2. Protein networks from the STRING database between OGT and OGA, kinases, and phosphatases (PDF)

## §

School of Chemistry and Chemical Engineering, Liaoning Normal University, Dalian 116029, China
