# Supplementary material for: Deciphering O‑GlcNAc-Dependent Signaling Via Integrated Proteomics and Phosphoproteomics
Source: ACS Omega. 2026 Jun 4;11(23):34373–84. doi: 10.1021/acsomega.6c02310 (PMC13280846; doi:10.1021/acsomega.6c02310)
Supplement: Supplementary file 2 [file ao6c02310_si_002.pdf]

Deciphering O-GlcNAc-dependent signaling via integrated proteomics and phosphoproteomics

Ci Wu,[1,2,*] Chunyan Hou,[1] Xinyue Wang,[2] Yihan Peng,[1] Yao Lin,[2] Stephen W. Byers,[1] Huadong Pei,[1] Junfeng Ma[1,*]

[1] Department of Oncology, Lombardi Comprehensive Cancer Center, Georgetown University Medical Center, Washington DC 20007, USA

[2] School of Chemistry and Chemical Engineering, Liaoning Normal University, Dalian 116029, China

[*] Corresponding author, E-mail: junfeng.ma@georgetown.edu

wuci@lnnu.edu.cn

Table of Contents

**Description**	**Page No.**
**Supplementary methods**	S-3
**Figure S1**. Cellular component (CC) and Molecular function (MF) enrichment analysis were performed for proteins corresponding to the five clusters.	S-5
**Figure S2**. Protein networks from STRING database between OGT and OGA, kinases and phosphatases.	S-6
**Table S1**. List of quantified proteins of PANC-1 cells upon disruption of O-GlcNAc cycling with TMG or OSMI-1.	File type, Excel
**Table S2**. List of quantified phosphorylation sites of PANC-1 cells upon disruption of O-GlcNAc cycling with TMG or OSMI-1.	File type, Excel
**Table S3**. List of classified phosphoproteins with dysregulated phosphosites identified after OSMI-1 or TMG treatment, grouped into five major clusters.	File type, Excel
**Table S4.** List of phosphorylation patterns using the motif-x algorithm of MoMo.	File type, Excel
**Table S5**. List of overlapped modification sites.	File type, Excel
**Table S6**. List of kinases with sites-specific phosphorylation changes after TMG or OSMI-1 treatment.	File type, Excel
**Table S7.** List of phosphatases with sites-specific phosphorylation changes after TMG or OSMI-1 treatment.	File type, Excel

Supplementary methods

Chemicals and Reagents

Trypsin, DTT (1,4-dithiothreitol), pyrrolidine, ammonium hydroxide solution (v/v, ~28%), phosphotase inhibitor, PUGNAC, benzonase, and glycolic acid were purchased from Sigma-Aldrich (St. Louis, MO). Triethylammonium bicarbonate buffer (1 M, pH 8.4-8.6), $MgCl_2$ was ordered from Fluka. Sodium cyanoborohydride ($NaCNBH_3$) were obtained from Alfa Aesar. Iodoacetamide (IAA) were ordered from VWR. Trifluoroacetic Acid (TFA) and acetonitrile (ACN, LC/MS grade) were purchased from Fisher Scientific (Waltham, MA). S-Trap midi columns were purchased from Protifi LLC (Farmingdale NY). Titansphere Phos-Tio Kit (TiO_2) was purchased from GL Sciences, Inc. (Tokyo, Japan). NanoAcquity UPLC mobile phase A: 0.1% formic acid in 2% ACN (LC/MS grade) and mobile phase B: 0.1% formic acid in ACN (LC/MS grade) were ordered from Honeywell. Protease inhibitor cocktail tablets were purchased from Roche (Mannheim, Germany). Thiamet G, Silmitasertib and OMSI-1 was obtained from Cayman Chemicals. Sequencing grade porcine trypsin was received from Promega (Madison, WI, USA). Dulbecco's Modified Eagle's medium (DMEM) was obtained from VWR chemicals.

Cell Culture

The pancreatic ductal cell line PANC-1 cells were grown in Dulbecco's modified Eagle's medium (DMEM) supplemented with 10% fetal bovine serum (FBS) and 1% penicillin/streptomycin. Cells were maintained in a 37 °C incubator with 5% CO_2. Cells were incubated with 2 μM Thiamet G, 2 μM OSMI-1, 2 μM silmitasertib or DMSO for 4 h before harvesting. After washing once with 10 mL cold PBS, cells were treated with 0.25% (w/v) trypsin/EDTA treatment. The cell suspension was centrifuged at 1200 rpm for 2 min at 4 °C, with the cell pellet kept at -80 °C before analysis.

Protein extraction and digestion

PANC-1 cell pellets were suspended in 200 µL cell lysis buffer (5% SDS, 2 µM PUGNAc, 1x protease inhibitor cocktail, 50 mM TEABC) by pipetting up and down. Benzonase (500 U) was added, with the lysate incubated on ice for 20 min. The cell suspension was then sonicated with a probe-tip sonicator for 5 pulses (10 sec on and 20 sec off for each pulse) on ice. The cell lysates were centrifuged at 13000 g for 15 min at 4 °C, with the supernatant transferred into a new 1.5 mL tube. Extracted proteins (300 µg) were processed with the suspension trapping (S-Trap) method as reported previously.[1] In brief, proteins were first reduced in 20 mM DTT by heating at 95 °C for 10 min. After cooling to room temperature, iodoacetamide was added to a final concentration of 40 mM for alkylation in darkness at room temperature for 30 min. The cell lysate solution was acidified by aqueous phosphoric acid (a final concentration of ~1.2% phosphoric acid) and diluted by six volumes of the S-Trap buffer (90% aqueous methanol in 100 mM TEABC, pH 7.1). The acidified mixture was transferred onto a midi S-Trap column followed by centrifugation at 2000 g for 1 min. After washing with the S-Trap buffer three times, proteins on the S-Trap column were digested with trypsin (an enzyme to substrate ratio of 1:50, w/w) at 37 °C overnight. The resulting peptides were eluted by adding 500 µL of 0.2% formic acid and 50% acetonitrile containing 0.2% formic acid sequentially. The eluants were combined and dried down with SpeedVac.

Bioinformatics

KinMap database (http://www.kinhub.org/kinmap/) was used with input of protein accession number from GPS library. The kinase families listed included TK (tyrosine kinases), TKL (tyrosine kinase-like), CK1 (casein kinase 1), CAMK (calcium/calmodulin-dependent protein kinase), AGC (containing PKA, PKG, PKC families), CMGC (containing CDKs, MAPK, GSK, CLK families), and STE (serine/threonine kinases many involved in MAPK kinases cascade).

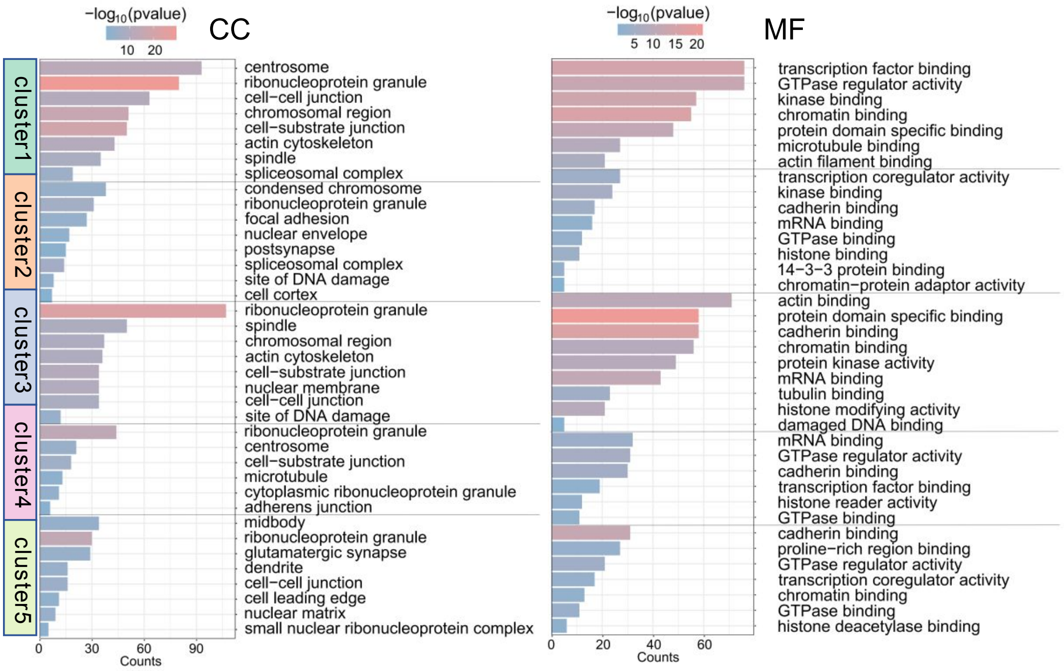

Figure S1. Cellular component (CC) (left) and Molecular function (MF) (right) enrichment analysis were performed for proteins corresponding to the five clusters.

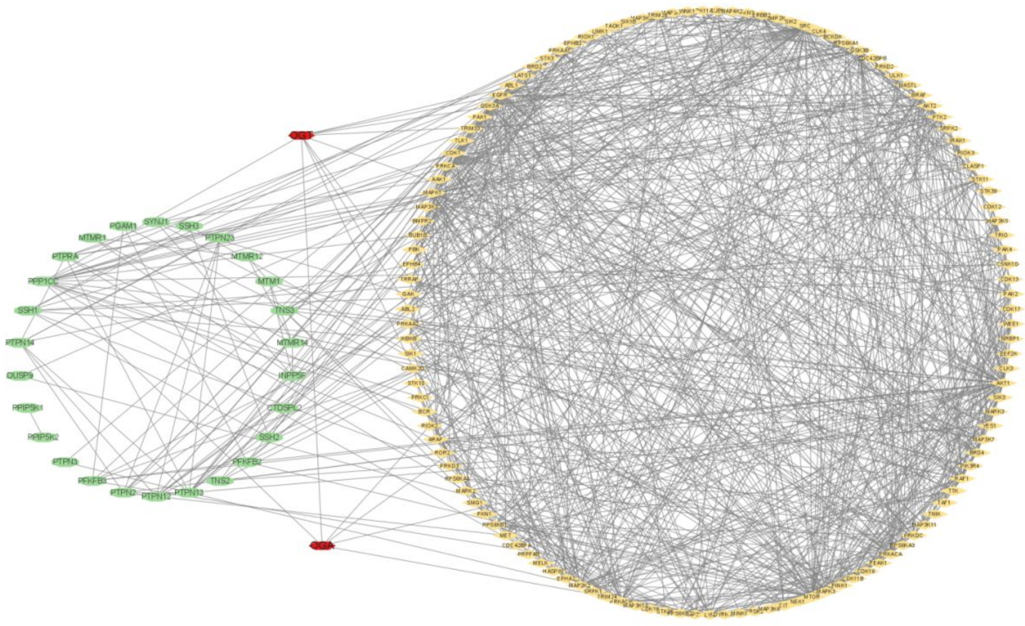

Figure S2. Protein networks from STRING database between OGT and OGA (red node), kinases (yellow node) and phosphotases (green node) with the dysregulated phosphosites upon treatment with TMG and OSMI-1.

References

[1] C. Wu, S. Zhou, M. I. Mitchell, C. Hou, S. Byers, O. Loudig, J. Ma, Coupling Suspension Trapping–Based Sample Preparation and Data-Independent Acquisition Mass Spectrometry for Sensitive Exosomal Proteomic Analysis. *Anal Bioanal Chem* **2022**, *414*, 2585.
